# Neuroprotective Effect of 1,4-Naphthoquinones in an *In Vitro* Model of Paraquat and 6-OHDA-Induced Neurotoxicity

**DOI:** 10.3390/ijms22189933

**Published:** 2021-09-14

**Authors:** Ekaterina Menchinskaya, Ekaterina Chingizova, Evgeny Pislyagin, Galina Likhatskaya, Yuri Sabutski, Dmitry Pelageev, Sergei Polonik, Dmitry Aminin

**Affiliations:** 1 G.B. Elyakov Pacific Institute of Bioorganic Chemistry, Far-Eastern Branch of the Russian Academy of Science, 690022 Vladivostok, Russia; martyyas@mail.ru (E.C.); pislyagin@hotmail.com (E.P.); galin@piboc.dvo.ru (G.L.); alixar2006@gmail.com (Y.S.); pelageev@piboc.dvo.ru (D.P.); sergpol@piboc.dvo.ru (S.P.); daminin@piboc.dvo.ru (D.A.); 2 School of Natural Sciences, Far Eastern Federal University, Ajax Bay, Russky Island, 690922 Vladivostok, Russia; 3 Department of Biomedical Science and Environmental Biology, Kaohsiung Medical University, Kaohsiung City 80708, Taiwan

**Keywords:** 1,4-naphthoquinones, neuronal cells, paraquat, 6-hydroxydopamine, cell viability, reactive oxygen species and nitric oxide production, mitochondrial membrane potential, cell cycle, Parkinson’s disease, neurotoxicity, neuroprotection

## Abstract

Targeted screening using the MTT cell viability test with a mini-library of natural and synthetic 1,4-naphthoquinones and their derivatives was performed in order to increase the survival of Neuro-2a neuroblastoma cells in in vitro paraquat and 6-hydroxydopamine models of Parkinson’s disease. As a result, 10 compounds were selected that could protect neuronal cells from the cytotoxic effects of both paraquat and 6-hydroxydopamine. The five most active compounds at low concentrations were found to significantly protect the activity of nonspecific esterase from the inhibitory effects of neurotoxins, defend cell biomembranes from lytic destruction in the presence of paraquat and 6-hydroxydopamine, and normalize the cell cycle. The protective effects of these compounds are associated with the suppression of oxidative stress, decreased expression of reactive oxygen species and nitric oxide formation in cells and normalization of mitochondrial function, and restoration of the mitochondrial membrane potential altered by neurotoxins. It was suggested that the neuroprotective activity of the studied 1,4-NQs is attributable to their pronounced antioxidant and free radical scavenging activity and their ability to reduce the amount of reactive oxygen species formed by paraquat and 6-hydroxydopamine action on neuronal cells. The significant correlation between the neuroprotective properties of 1,4-naphthoquinones and Quantitative Structure–Activity Relationship descriptors describing the physicochemical properties of these compounds means that the hydrophobicity, polarity, charge, and shape of the molecules can be of decisive importance in determining the biological activity of studied substances.

## 1. Introduction

Parkinson’s disease (PD) is an age-dependent and slowly progressive chronic neurodegenerative disease. It refers to a degenerative disease of the extrapyramidal motor system. PD is caused by the progressive destruction and death of neurons that produce the neurotransmitter dopamine, primarily not only in the substantia nigra but also in other parts of the central nervous system. It is the second most common neurodegenerative disease worldwide [1,2]. Although the exact cause of this disease is still unclear, it is believed that exposure to environmental neurotoxins is one of the decisive factors in its aetiology.

Paraquat (PQ) is *N*,*N*′-dimethyl-4,4′-dipyridylium dichloride, a highly toxic compound used in a number of countries as a potent herbicide. PQ was one of the first compounds used to model PD. The neurotoxic properties of PQ are primarily mediated by its effect on the redox cycle and the functioning of the nitric oxide synthase enzyme in neuronal cells. This, in turn, induces the increased production of reactive oxygen species (ROS) and increases the levels of α-synuclein and tau protein, α-tubulin hyperacetylation, inhibition of proteasomes, and dysfunction of axonal autophagy, which result in symptoms of PD [3].

6-Hydroxydopamine (6-OHDA) is another neurotoxic compound that is often used as an inducer of neurodegenerative changes in an in vitro model of PD. It is a structural analogue of dopamine, which explains its selective uptake into dopaminergic and noradrenergic neurons via a membrane dopamine transporter and a norepinephrine transporter, respectively. In neurons, 6-OHDA accumulates in the cytosol, where, with the participation of the enzyme monoamine oxidase (MAO), it is metabolized into dihydrophenylacetic acid or oxidized to form hydrogen peroxide and para-quinone, which results in ROS formation and oxidative stress in neurons with their subsequent death [3].

Both of these PD inducers, along with two other neurotoxins, rotenone and MPTP (1-methyl-4-phenyl-1,2,3,6-tetrahydropyridine), are widely used in experiments in order to study the neuroprotective properties of natural and synthetic compounds. However, despite the intense search for a cure for PD, potent and targeted medicines have not yet been developed, which confirms the need to study new selective and effective drugs.

1,4-Naphthoquinones (1,4-NQs) are important metabolites in plants and animals. Their natural structures and synthetic derivatives demonstrate a wide spectrum of cytoprotective activities. These compounds have been found to exhibit antibacterial, antifungal, antioxidant, hepatoprotective, cardioprotective, anti-ischaemic properties and other properties. Their role in protecting against neurodegenerative diseases such as Alzheimer’s disease (AD) and Parkinson’s disease has also been established [4,5,6,7,8,9,10,11,12]. One of the 1,4-NQs synthesized by our group, U-133, exhibited a promising neuroprotective effect in an in vivo PD rat model [13].

Recently, we synthesized a library of 5,8-dihydroxy-1,4-naphthoquinone derivatives (44 compounds) and studied their cytotoxic properties against mouse neuronal Neuro-2a cells using the QSAR approach [14]. In the present study, we evaluated whether these 1,4-NQ derivatives protect Neuro-2a cells and increase cell viability in two in vitro models of PD induced by the PQ and 6-OHDA neurotoxins. We assessed the capability of 1,4-NQs to inhibit ROS and NO generation in cells under neurotoxin treatment, their effect on biomembrane permeability, the cell cycle and mitochondrial membrane potential, and inspected their radical scavenging activity.

## 2. Results

### 2.1. Preparation of 1,4-NQ Derivatives

A library of 44 natural and synthetic 1,4-NQ derivatives tested in the present study was developed in our previous work [14]. Briefly, four groups of NQ compounds were tested: a group of basic NQs (sea urchin pigments and its methoxy derivative); NQ acetylated O-glucosides; NQ acetylated S-glucosides; and NQ deacetylated S-glucosides. The last two groups were constructed by using thiomethylation of 6,7-substituted 2,5,8-trihydroxy-1,4-naphthoquinones with paraformaldehyde and per-O-acetyl-1-thioglucose. Methylation of the 2-hydroxy group of the quinone core of acetylthiomethylglycosides by diazamethane and deacetylation of the sugar moiety resulted in 28 new thiomethylglycosides of 2-hydroxy-1,4-NQs and 2-methoxy-1,4-NQs. In total, 44 compounds were applied and assayed in the present research. The chemical structures of the studied 1,4-NQ derivatives are shown in Table 1. The cytotoxic activities (EC_50_) for most compounds, except **U-642** and **U-643** against mouse neuronal Neuro-2a cells, were established in our earlier investigation [14] and are also included in Table 1.

### 2.2. Determination of the Cytoprotective Properties of 1,4-NQs in An In vitro PD Model Induced by PQ and 6-OHDA

#### 2.2.1. Effect of 1,4-NQs on Cell Viability Using MTT Assay

**Figure 1:**
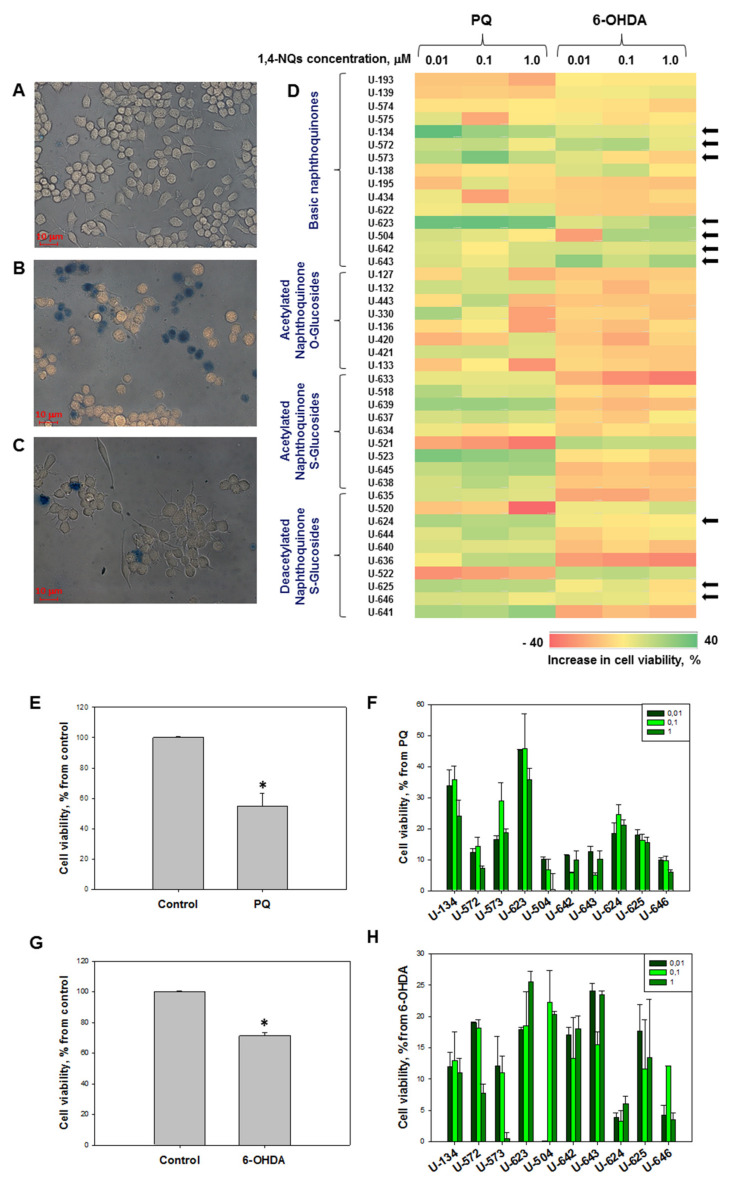
1,4-NQs protect Neuro-2a cells against the neurotoxicity of PQ and 6-OHDA. (**A**) control cells; (**B**) cells cultivated in the presence of 6-OHDA (90.0 μM) for 24 h; (**C**) cells pretreated with U-623 (1.0 μM) and cultivated in the presence of 6-OHDA for 24 h. After incubation, the cells were stained with Trypan blue dye. (**D**) heat map visualization of the influence of 1,4-NQs on the viability of Neuro-2a cells cultivated in the presence of neurotoxins, PQ or 6-OHDA. Data are normalized to the viability of cells treated with neurotoxins alone (negative control). Arrows indicate the compounds protecting cell viability in the presence of PQ and 6-OHDA. (**E**,**G**) quantification of the effect of PQ (1.0 mM) and 6-OHDA (90.0 μM) on cell viability. (**F**,**H**) quantification of the impact of 1,4-NQs on the viability of cells incubated in the presence of PQ or 6-OHDA. Cell viability was evaluated by the MTT method. Data are presented as m ± se (*n* = 3). * *p* < 0.05 compared to cells exposed to PQ or 6-OHDA alone.

**Figure A1:**
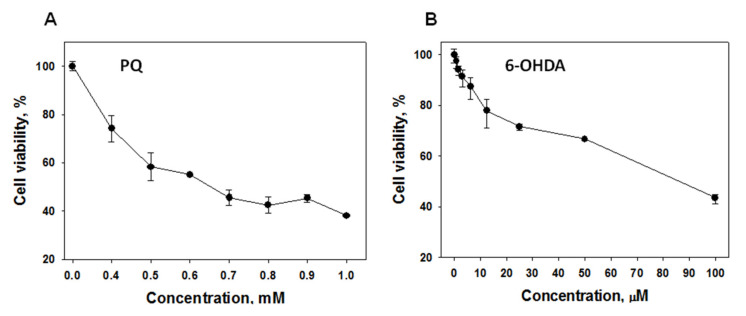
Cytotoxic effect of neurotoxins, PQ (**A**) and 6-OHDA (**B**), on Neuro-2a cell viability. Neuro-2a cells (1 × 10^4^ cells/well) were incubated in a CO_2_ incubator for 24 h at 37 °C for adhesion. After that, 20 μL of neurotoxin test solution at different concentrations was loaded to the cells and incubated for 24 h. Then, cell viability was determined by using MTT assay. Data are presented as m ± se (*n* = 3).

In order to determine the cytoprotective (neuroprotective) properties of 1,4-NQ derivatives in a PD model with mouse neuroblastoma Neuro-2a cells induced by PQ or 6-OHDA, neurotoxins were introduced into the culture medium at concentrations that caused approximately 50% cell death (Figure 1A–C; Appendix A, Figure A1). All quinone compounds in the noncytotoxic concentration range of 1 μM and below were added to the cell suspension 1 h before application of the PD inducer. Then, the cells were incubated for one day, and the viability of the cells was determined. Based on the data obtained, compounds that were able to protect cells from the damaging effects of neurotoxins and significantly increase their viability in the presence of neurotoxins were selected.

The general data obtained show that the studied 1,4-naphthoquinones are capable of protecting neuronal cells from the cytotoxic action of neurotoxins but not to the same extent. The presented heat-map visualization of data on the effects of the studied 1,4-NQs on the increase in cell viability in the presence of neurotoxins indicates that the studied compounds are, in the overwhelming majority of cases, able to protect neuronal cells from PQ cytotoxic action (Figure 1D). Thus, the number of compounds increasing Neuro-2a cell viability up to 40% in the presence of PQ (coloured in green) is much higher compared to cells incubated in the presence of 6-OHDA.

Nevertheless, a number of 1,4-NQs exhibited cytoprotective properties in almost the same manner in both PD models in vitro, both during cell cultivation with PQ and with 6-OHDA. There were 10 compounds, **U-134**, **U-572**, **U-573**, **U-623**, **U-504**, **U-642**, **U-643**, **U-624**, **U-625**, and **U-646**. The quantified cytoprotective effects of these compounds are presented in Figure 1. As shown in Figure 1E,G, the neurotoxins PQ and 6-OHDA alone caused decreases in cell viability by approximately 40–50%. The studied 1,4-NQs reliably protected cells from the neurotoxic effects of PQ. The highest neuroprotective activity was shown by substances **U-134**, **U-573**, **U-623**, and **U-624** at concentrations of 0.01 μM and 0.1 μM, respectively. At a concentration of 0.1 μM, **U-134** protected neuronal cells from PQ-damaging effects and significantly increased the number of viable cells by 35.7%; substance **U-573** increased this parameter by 28.8%, while compounds **U-623** and **U-624** increased cell viability by 45.7% and 24.6%, respectively, compared to control cells exposed to PQ alone (Figure 1F).

Ten selected compounds significantly protected cells from the neurotoxic effect of 6-OHDA by approximately 8–27%. The greatest effects were shown for compounds **U-134**, **U-624**, **U-572**, **U-504**, and **U-643**. These substances at a concentration of 0.1 μM increased the viability of 6-OHDA-exposed neuronal cells by 12.9%, 18.5%, 18.3%, 22.3%, and 15.5%, respectively (Figure 1H).

#### 2.2.2. Effects of 1,4-NQs on Cell Viability in FDA and PI Tests

**Figure 2:**
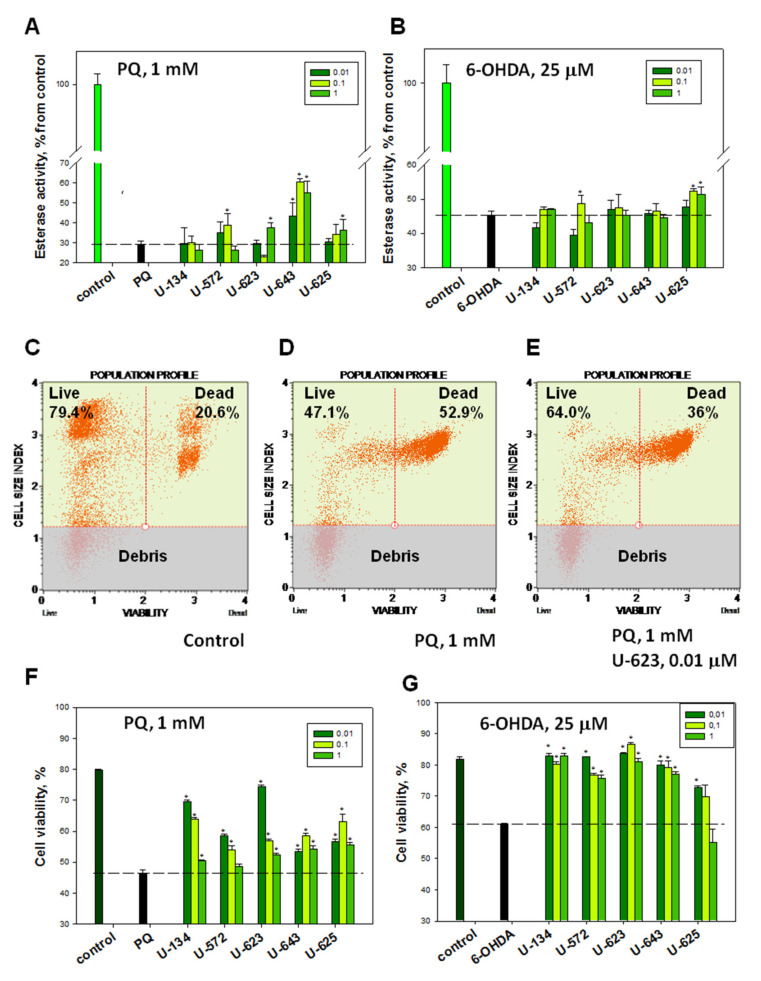
1,4-NQs protect Neuro-2a cells against neurotoxicity induced by PQ and 6-OHDA. (**A**,**B**) determination of nonspecific esterase activity in cells incubated with PQ (1.0 mM, **A**) or 6-OHDA (25.0 μM, **B**) using a FDA fluorescent probe and spectrofluorimetry. (**C**–**E**) count and viability assay. The figures show the percentage of live and dead cells determined using flow cytometry and PI staining: Control (**C**), PQ, 1.0 mM (**D**); PQ, 1.0 mM, and **U-623**, 0.01 μM (**E**). (**F**,**G**) determination of the viability of cells incubated with PQ (**F**) or 6-OHDA (**G**) using a PI fluorescent probe and fluorescent flow cytometry. Concentrations of 1,4-NQs in the culture medium were 0.01, 0.1, or 1.0 μM. Data are presented as m ± se (*n* = 3). * *p* < 0.05 compared to cells exposed to PQ or 6-OHDA alone.

The protective properties of 1,4-NQs determined by the MTT test were examined by several other independent methods. It was found that the PD inducers 6-OHDA and PQ inhibited intracellular nonspecific esterase, the activity of which is a known indicator of cell viability. The five selected most effective 1,4-NQs exhibiting cytoprotective properties on neuroblastoma cells in the MTT test also demonstrated cytoprotective activity in the test determining esterase activity using an FDA fluorescent probe. Thus, compounds **U-134**, **U-572**, **U-643**, and **U-625** at a concentration of 0.1 μM protected the activity of nonspecific esterase from the PQ-damaging effect by 2.8, 32.6, 107.7, and 17.4%, respectively (Figure 2A). Similar results were obtained with the same 1,4-NQs in the test where 6-OHDA was used as a neurotoxin. Compounds **U-134**, **U-572**, **U-623**, **U-643**, and **U-625** were found to prevent and recover cell nonspecific esterase activity in the presence of 6-OHDA by 3.4%, 7.3%, 4.5%, 2.6%, and 14.9%, respectively (Figure 2B).

The effect of 1,4-NQs on the viability of neuronal cells under the toxic effect of PD inducers in vitro was studied by staining cells with the fluorescent dye PI. The intensity of the fluorescence of this probe due to its binding to DNA characterizes dead cells in which biomembrane permeability is impaired. Using a fluorescent flow cytometry approach, it was shown that PQ (Figure 2D,F) and 6-OHDA (Figure 2G) caused significant increases in the number of PI-stained cells in the population and, therefore, increases in the percentage of dead cells. The most effective 1,4-NQs, having a cytoprotective effect in the MTT test and in the test with nonspecific esterase, also exhibited defensive properties in the PI test. Thus, compounds **U-134**, **U-572**, **U-623**, **U-643**, and **U-625** defended biomembrane permeabilities from the damaging effects of PQ by 37.0%, 16.0%, 22.4%, 25.4%, and 35.8% (Figure 2F), while the same compounds preserved biomembrane integrity in the presence of 6-OHDA by 31.3%, 25.4%, 41.6%, 29.5%, and 14.1%, respectively (Figure 2G).

### 2.3. Influence of 1,4-NQs on Neuro-2a Cell Cycle Disturbance Caused by PQ and 6-OHDA

**Figure 3:**
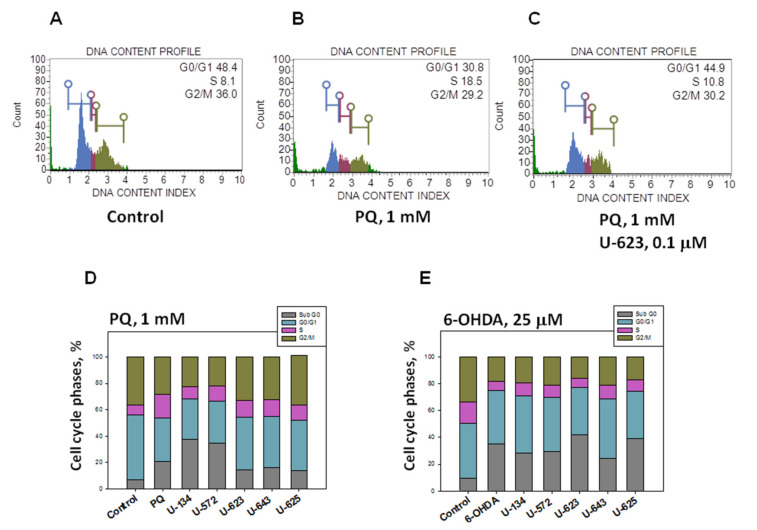
Effect of 1,4-NQs on the Neuro-2a cell cycle altered by PQ and 6-OHDA. Cells (1.5 × 10^5^ cells/well in 6-well plates) were incubated with PQ (1.0 mM) or 6-OHDA (25.0 μM) for 24 h. The cell cycle was analysed using fluorescent flow cytometry. (**A**–**C**) the figure shows the percentage of cells in each stage of the cell cycle: Control (**A**), PQ, 1.0 mM (**B**), PQ, 1.0 mM, and U-623, 0.1 μM (**C**). (**D**,**E**) histogram of cell cycle distribution (%) in Neuro-2a cells exposed to PQ (**D**) or 6-OHDA (**E**) and treated with different 1,4-NQs at a concentration of 0.1 μM for 24 h. Values are expressed as the means of three different experiments.

In order to elucidate whether the mechanism of reduction and recovery in cell viability also involves cell cycle changes, the effects of 1,4-NQs on Neuro-2a cell cycle progression in the presence of neurotoxins were determined by flow cytometry. PQ and 6-OHDA were found to alter the cell cycle. PQ arrested the cell cycle in DNA replication phase S and consequently decreased in the resting or initial G0/G1 and mitotic G2/M phases (Figure 3B,D). 6-OHDA induced a decrease in the cell population in the S and G2/M phases but did not significantly influence the G0/G1 phase (Figure 3E). Both neurotoxins evoked notably increased haploid/apoptotic cell numbers in the sub-G0 phase.

The most effective 1,4-NQs supporting cell survival in the MTT, esterase, and PI tests also contributed to the normalization of the cell cycle of cells exposed to neurotoxins. The increase in the PQ nonproliferating cell population with compounds **U-134**, **U-572**, **U-623**, **U-643**, and **U-625** at a concentration of 0.1 μM was accompanied by a reciprocal decrease in cells in S-phase by 47.3, 36.3, 29.9, 27.7, and 40.9%, respectively (Figure 3D). At the same time, compounds **U-134**, **U-572**, and **U-643** most effectively and significantly recovered the cell population in the S and G2/M phases in the presence of 6-OHDA by 41.2%, 32.6%, and 52.5% and by 7.5%, 16.1%, and 15.9%, respectively (Figure 3E). Almost all compounds reduced the haploid/apoptotic cell number, except **U-134** and **U-572**, which surprisingly increased this index slightly in PQ-treated cells.

### 2.4. Evaluation of ROS and NO Production Levels in Neuro-2a Cells Caused by PQ and 6-OHDA in the Presence of 1,4-NQs

The most active 1,4-NQs selected in the previous test were studied for their ability to suppress the oxidative burst in neuronal cells caused by PQ and 6-OHDA. For this purpose, compounds in the noncytotoxic concentration range were incubated with cells 1 h before the inducer was introduced into the culture. Then, the cells were incubated for 1 h with PD inducers at a concentration that caused 40–60% suppression of cell viability. After that, ROS and NO levels in the cells were determined spectrofluorimetrically.

**Figure 4:**
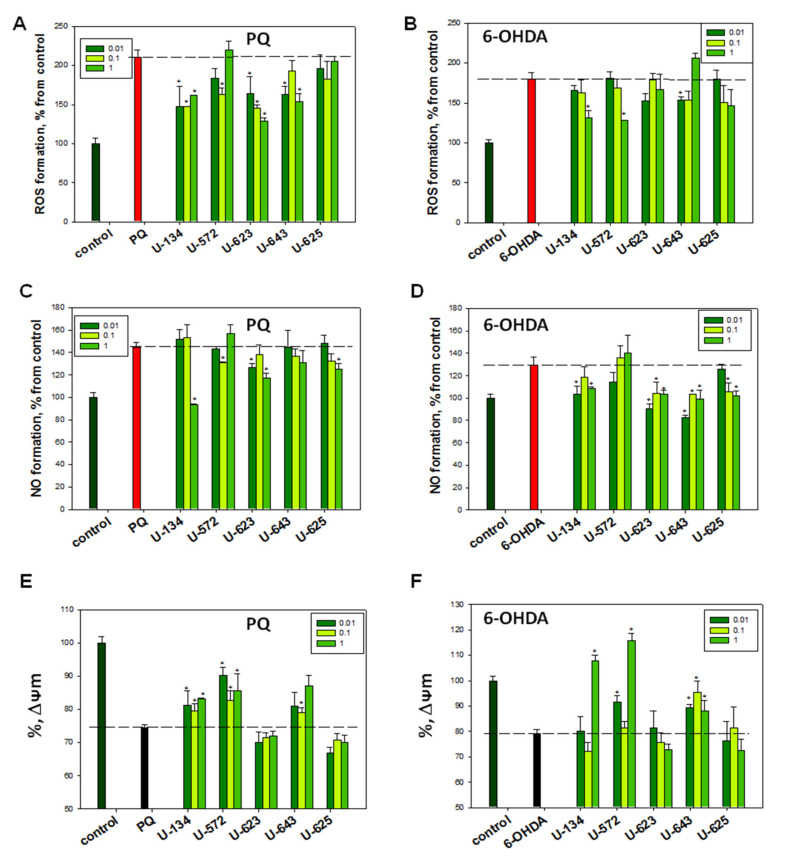
The effects of 1,4-NQs on the levels of ROS and NO production and MMP levels in Neuro-2a cells. Cells (1 × 10^4^ cells/well) were incubated with PQ at 1.0 mM for 3 h (**A**,**C**); or for 1 h (**E**); or 6-OHDA, 25.0 μM for 3 h (**B**,**D**); or 90.0 μM for 5 h (**F**). ROS, intracellular NO levels and MMP levels were measured with *H_2_DCF-DA,* DAF-FM and TMRM fluorescent probes using spectrofluorimetry. Data are presented as m ± se (*n* = 3). * *p* < 0.05 compared to cells exposed to PQ or 6-OHDA alone.

As shown in Figure 4, both neurotoxins, PQ and 6-OHDA, induced noticeable amplification of ROS and NO production in neuronal cells. Four substances, **U-134**, **U-572**, **U-623**, and **U-643**, were found to significantly reduce ROS formation in the cells exposed to paraquat. The most powerful effect was exerted by substances **U-134** and **U-623** at a concentration of 0.1 μM. They reduced the amount of ROS in the cells by 30.2% and 31.0%, respectively. Substances **U-572** and **U-643** were less active, while **U-625** was ineffective at all studied concentrations (Figure 4A).

Preincubation of Neuro-2a cells with selected 1,4-NQs reduced NO production in PQ-exposed cells. The most pronounced effect was observed for **U-134** at a concentration of 1.0 μM; it amounted to approximately 35.4%. Substances **U-623** (1.0 μM) and **U-625** (0.1 μM) were less effective and showed the ability to decrease NO levels in PQ-treated cells by 17.0% and 13.6%, respectively. Compounds **U-572** and **U-643** were completely ineffective at all studied concentrations (Figure 4C).

Based on the results obtained, only three compounds were selected that significantly diminished ROS production in 6-OHDA-treated neuronal cells. **U-134** (1.0 μM), **U-572** (1.0 μM), and **U-643** (0.01 μM) decreased ROS levels by 31.6%, 28.6% and 10.6%, respectively (Figure 4B).

At the same time, four studied 1,4-NQs, **U-134**, **U-623**, **U-643**, and **U-625**, significantly reduced NO production in 6-OHDA-treated cells. These compounds significantly mitigated NO levels in cells by approximately 3–40%, depending on the concentration (Figure 4D). The maximal effect was observed for compound **U-643**, which reduced nitric oxide production in cells by approximately 36.4% at a concentration of 0.01 μM. Compound **U-572** did not exhibit any perceptible decrease in NO levels in cells exposed to 6-OHDA alone (Figure 4D).

### 2.5. Influence of 1,4-NQs on Mitochondrial Membrane Potential Altered by PQ and 6-OHDA

Incubation of neuroblastoma cells with PQ and 6-OHDA resulted in a displacement charge on the mitochondrial membrane potential (MMP), expressed as a decrease in the fluorescence intensity of the specific molecular probe TMRM (depolarization). It was found that some of the studied 1,4-NQs are able to prevent depolarization and restore MMP to almost baseline values. The most active naphthoquinones that protected MMP in the case of both PQ and 6-OHDA applications were **U-134**, **U-572**, and **U-643**, while **U-623** and **U-625** were virtually inactive. The most effective 1,4-naphthoquinone was **U-134** and **U-572**, which, at a concentration of 0.01 μM, increased the MMP values by 8.9% and 21.2% compared to the action of PQ alone, respectively. In the case of 6-OHDA application, Compounds **U-134** and **U-572** caused even slight hyperpolarization, expressed as increased TMRM probe fluorescence compared to the values of control neurotoxin-untreated cells (Figure 4E,F).

### 2.6. Effects of 1,4-NQs on Scavenging DPPH Radical

The free radical scavenging activity of the tested 1,4-NQs was investigated using the DPPH assay approach. Ascorbic acid was chosen as a control compound with pronounced antioxidant and free radical scavenging activity. The scavenging activity of the tested 1,4-NQs at concentrations of 0.01 μM and 0.1 μM was significantly and reliably higher than that of ascorbic acid used at the same concentrations. Thus, compounds **U-134**, **U-572**, and **U-623** at nanomolar concentrations were almost 3–4 times more active in DPPH radical scavenging than ascorbic acid. At the same time, the ability of naphthoquinones to capture free radicals at a concentration of 1.0 μM was higher but, in fact, comparable to that of ascorbic acid (Table 2).

### 2.7. Generation and Validation of QSAR Models

QSAR analysis was performed on neuroprotection data (Table 1 and Figure 1) derived from active basic 1,4-NQs in PD models in Neuro-2a cells induced by PQ and 6-OHDA neurotoxins. For neuroprotection data using PQ or 6-OHDA as an inducer, two separate datasets were created. Compounds included for calculations in the PQ and 6-OHDA datasets were as follows:

PQ: (**U-642**, **U-643**, **U-622**, **U-504**, **U-195**, **U-572**, **U-134**, **U-573**, and **U-623**);

6-OHDA: (**U-139**, **U-134**, **U-623**, **U-138**, **U-572**, **U-504**, **U-642**, and **U-643**).

The 320 descriptors were calculated by the MOE QuaSAR-Model module for each molecule in the datasets. The calculated descriptors were initially screened using the QuaSAR-Contingency module of MOE, which is a statistical application designed to assist in the selection of descriptors for QSAR. The QuaSAR-Model module of MOE was used to create QSAR models with the “partial least square” (PLS) method.

Analysis of the models using the QuaSAR-Model report made it possible to select a smaller number of the most important molecular descriptors and obtain models with a smaller number of descriptors. The descriptors with an effect on predicting the performance of neuroprotective activity of 1,4-NQs with QSAR models and used for QSAR model generation are described in Table 3. The best QSAR estimated linear models for neuroprotection are as follows.
PQ = 6-OHDA =68.61008 44.40310+2.63075 * SlogP_VSA3 +0.48187 * SMR_VSA7+2.63075 * SMR_VSA2 −0.31397 * SlogP_VSA0−4.17081 * TPSA −0.33998 * SlogP_VSA5−2.29788 * SMR_VSA3 −0.34822 * SlogP_VSA9+4.32894 * SlogP_VSA0 +0.31683 * TPSA+6.35996 * SlogP_VSA6 −0.10669 * vsurf_D1+0.82448 * SlogP_VSA5 


Analysis of the relative importance of QSAR descriptors for the neuroprotective activity of 1,4-NQs showed that the most important descriptors are the following:

PQ: (TPSA, SlogP_VSA0, SlogP_VSA3, and SMR_VSA2);

6-OHDA: (TPSA, SlogP_VSA0, SMR_VSA7, and vsurf_D1).

**Figure 5:**
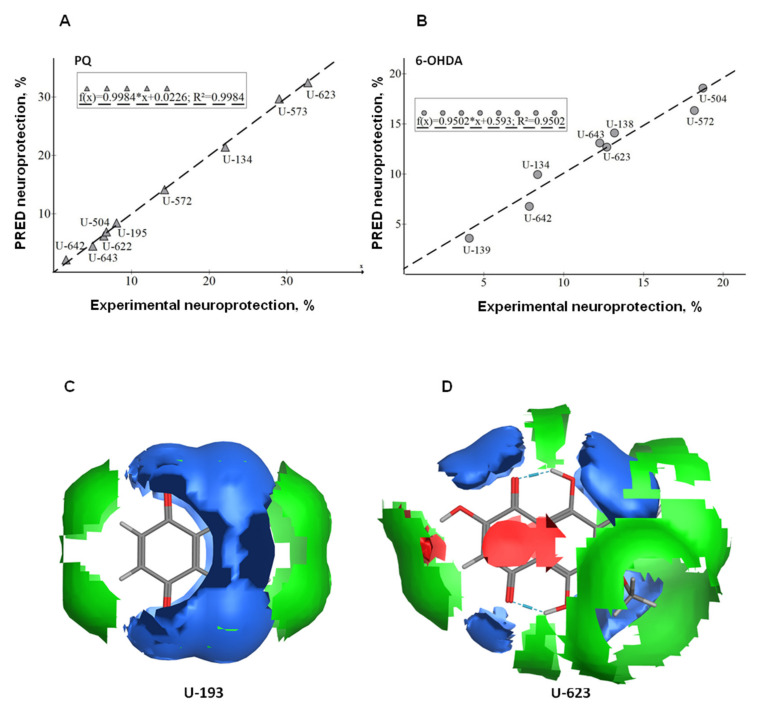
Predicted neuroprotective activity (%) of basic 1,4-NQs (0.1 μM) as a function of experimental values for the Neuro-2a cell line in the presence of PQ (**A**) or 6-OHDA; (**B**) the electrostatic feature map predicting electrostatically preferred locations of hydrophobic, H-bond acceptor, and H-bond donor locations for non-active **U-193** (**C**) and highly active neuroprotective **U-623** (**D**). Data on experimental cytoprotective activity were obtained using the MTT method (Figure 1). The colour value for the isocontour surface of a particular interaction atom type is green—hydrophobic; blue—donor; red—acceptor interaction atom types. The maps are displayed for the default energy values (in kcal/mol) of −0.4 for the hydrophobic map and −2 for H-bond acceptor and H-bond donor maps.

QSAR models were constructed based on the selected molecular descriptors using the QuaSAR-Model module in MOE 2020.09. QuaSAR-Model regression analysis was used to build a QSAR model using PLS. The samples of compounds with neuroprotective activity consisted of nine compounds for the PQ inducer and eight compounds for the 6-OHDA inducer for which the models were constructed. For the PQ group, a model was obtained with a correlation coefficient (R2) of 0.9984. In the case of 6-OHDA, a model was obtained with a correlation coefficient (R2) = 0.9502 (Figure 5A,B).

Additionally, prediction of electrostatically preferred locations of hydrophobic, H-bond acceptor, and H-bond donor locations in two basic 1,4-naphthoquinone molecules, non-active **U-193** and highly active neuroprotector **U-623**, was made using the electrostatic feature map approach based on the Poisson–Boltzmann equation (PBE) application. Figure 5C,D indicate the generated predictive surface electrostatic maps of 1,4-NQs, where the hydrophobic map is an isocontour of the van der Waals potential of a carbon atom (green colour), the acceptor map is an isocontour of the partial charge of the oxygen ion and the van der Waals potential of the oxygen atom (red colour), and the donor map is an isocontour of the partial charge of the hydrogen ion and van der Waals potential of the hydrogen atom (blue colour). This map suggests that the most active compound **U-623** is distinguished by a large hydrophobic area and the presence of acceptor sites in the molecule, while the absolutely inactive compound **U-193** is characterized by a significant contribution of donor sites to the overall picture of the electrostatic surface, a significantly smaller hydrophobic zone, and a practical lack of acceptor centres.

## 3. Discussion

Among 1,4-naphthoquinones and their derivatives, compounds with neuroprotective activity have already been found. Thus, some naphthoquinone derivatives, such as naphthoquinone-tryptophan, have been shown to inhibit α-synuclein (α-Syn) aggregation into toxic oligomers and mature fibrils, which is the major pathological hallmark of PD [5]. Some 1,4-naphthoquinone derivatives, such as 2,3,6-trimethyl-1,4-naphthoquinone present in tobacco leaves and 5,8-dihydroxy-1,4-naphthoquinone (naphthazarin), are reported to inhibit both forms of monoamine oxidases, MAO-A and MAO-B, with a reversible mode of action [6,7,8,9], which may specifically explain a certain level of the resistance of tobacco smokers to PD. More recently, it was reported that 2-methoxy-1,4-naphthoquinone stimulates the therapeutic neural repair properties of olfactory ensheathing cells [10]. In a model of PD in knockout rats, it was shown that the acetylated tris-O-glucoside of echinochrome A **U-133** activates the HSF1 transcription factor and increases the expression of inducible Hsp70, which results in a reversal of neurodegeneration processes [11]. It was later found that echinochrome A **U-138** is a noncompetitive acetylcholinesterase inhibitor, which opens up prospects for the use of this compound for the treatment of neuromuscular disorders, as well as Alzheimer’s and Parkinson’s diseases [12]. Recently, therapy based on **U-133** administration was applied in a model of the preclinical stage of PD in elderly rats. It was shown that **U-133** weakened the process of neurodegeneration in the substantia nigra pars compacta and counteracted the development of neuroinflammation, decreased the amount of aggregated α-synuclein and regression of post-translationally modified α-synuclein, eliminated anhedonia, and prevented the development of neurodegeneration in monoaminergic emetogenic structures of the brain in experimental PD [13,15]. Therefore, the study of the neuroprotective effects of 1,4-naphthoquinones in general and their antiparkinsonian activity in particular may have good prospects.

We tested the cytoprotective properties of our collection of 1,4-NQ derivatives presented in Table 1 and selected ten compounds capable of protecting neuroblastoma Neuro-2a cells based on the cytotoxic action of both neurotoxins, PQ and 6-OHDA. These ten 1,4-NQs are as follows: **U-134**, **U-504**, **U-572**, **U-573**, **U-623**, **U-624**, **U-625**, **U-642**, **U-643**, and **U-646**. In tests with neurotoxins, these NQs increased the viability of Neuro-2a cells to 30–40% at low concentrations. Among the ten compounds, the five most active 1,4-NQs were selected for further advanced studies of their neuroprotective properties. It has been shown that these substances protect the activity of the enzyme nonspecific esterase from the inhibitory effect of neurotoxins, protect cell biomembranes from lytic destruction in the presence of PQ and 6-OHDA, and normalize the cell cycle. The protective effect of these compounds was associated with the suppression of oxidative stress, expressed as a decrease in ROS and NO formation in cells and normalization of the functioning of mitochondria and restoration of the mitochondrial membrane potential altered by neurotoxins.

**Scheme 1:**
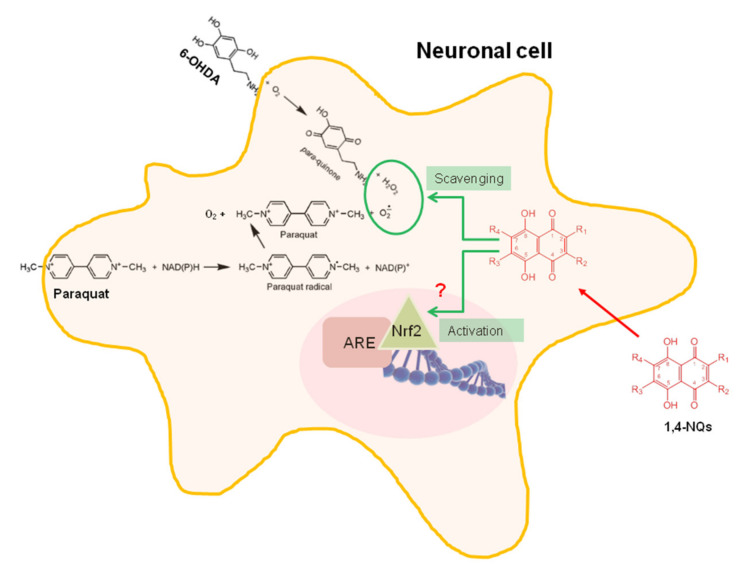
Hypothetical cytoprotective mechanism of 1,4-NQs against PQ-induced and 6-OHDA-induced neurotoxicity.

The molecular mechanisms of the neurotoxic action of PQ and 6-OHDA have already been studied in detail. These neurotoxins, although distinct in their intimate cytotoxic mechanisms, kill dopaminergic neurons via a cascade of deleterious events that consistently involves oxidative stress. Once in neuronal cells, paraquat undergoes two-stage oxidation–reduction cycling with the subsequent formation of superoxide radicals. In the first stage, the paraquat molecule is oxidized with the participation of diaphorase (mainly nitric oxide synthase in the brain) in order to form a paraquat cation, which at the second stage undergoes reoxidization to form oxidized paraquat and O_2_**^●−^** (Scheme 1). The superoxide radicals formed by PQ play a decisive role in the manifestation of its cytotoxicity and neurototoxicity [3,16]. Due to its structural similarity with dopamine and norepinephrine, the neurotoxin 6-OHDA penetrates dopaminergic and noradrenergic neurons and causes the destruction of the catecholaminergic pathways of the nervous system. The oxidation of 6-OHDA produces para-quinone and ROS such as H_2_O_2_, as well as superoxide and hydroxyl radicals (Scheme 1). In turn, ROS can directly or indirectly induce a number of cellular oxidation reactions, which ultimately result in neurotoxicity and neuronal cell death [16].

It is quite obvious that the formation of toxic ROS under the influence of PQ and 6-OHDA causes all other toxic effects in the Neuro-2a neuroblastoma cells we used, namely, the inhibition of activity of enzymes such as NADPH-dependent cellular oxidoreductase enzymes (reducing tetrazolium dye in the MTT test) and nonspecific esterases reflecting cell metabolic function, disturbance of the biomembrane integrity, violation of the cell cycle, alteration of mitochondrial function, and mitochondrial membrane potential.

In all likelihood, the studied 1,4-NQs exhibit their neuroprotective properties due to their antioxidant and free radical scavenging activities. The molecular mechanisms of antioxidant action have been most fully described for a number of natural polyhydroxy-1,4-naphthoquinones with naphthazarin (5,8-dihydroxy-1,4-naphthoquinone) cores. It has now been established that the high antioxidant activity of echinochrome A **U-138** and related polyhydroxynaphthazarins **U-134**, **U-572**, **U-573**, **U-504**, **U-642**, and **U-643** is due to their ability to intercept free radicals, in particular, superoxide anion radicals and peroxyl radicals [17]. Thus, the sea urchin pigment echinochrome A **U-138** is used in the Russian Pharmacopoeia as the medicine Histochrome™ for cardiology and ophthalmology and in the treatment of chronic lung diseases [17,18,19].

*β*-Hydroxy groups at positions 2, 3, and 7 of echinochrome A are responsible for quinone redox transformations, antioxidant properties, and biological activity. Methylation of *β*-hydroxyls in echinochrome A resulted in the formation of echinochrome trimethoxy derivatives completely devoid of antioxidant activity [17]. Hydroxynaphthoquinone antioxidants prevent the free radical oxidation process of hydrogen atom transfer and interrupt the oxidative chain through the interception (inhibition) of free radical particles [18,19,20,21].

1,4-NQ derivatives (Figure 1D) demonstrated the highest cytoprotective activity in the test with neurotoxin PQ. All active compounds marked by arrows in the basic NQ group are sea urchin pigments or the methoxyderivative **U-623**. These quinones are β-hydroxy-5,8-dihydroxy-1,4-naphthoquinone (naphthazarin) derivatives bearing readily oxidizable β-hydroxy groups in the naphthazarin core. Compounds **U-642** and **U-643** are spinochrome D **U-504** dimers linked through a methylene and ethylidene bridge. Surprisingly, echinochrome A **U-138** showed little protective effect against PQ and 6-OHDA among the tested sea urchin pigments.

Active NQs of this group show similarity with the vitamin C structure. Both include the double bond with hydroxyl groups conjugated with one (vitamin C) or two carbonyl groups (hydroxynaphthazarins) involved in five-member or six-member rings. This close similarity to vitamin C allows natural hydroxynaphthazarins to easily enter the cell, circulate there, and protect cell fine structures from toxin action.

Glucosidation of hydroxynaphthoquinones results in the blocking of all β-hydroxy groups through the formation of O-glucoside derivatives with a significant decrease in the protective properties of the quinone core. Acetylated O-glucosides of NQs were inactive in the 6-OHDA test and weakly protected Neuro-2a cells from the action of PQ. It seems that the conversion of NQs in the acetylated O-glycosides and S-glycosides results in a complete loss of the ability of these compounds to capture hydrogen peroxide, superoxide, and hydroxyl radicals formed under this neurotoxin treatment.

The highest cytoprotective activity of 1,4-naphthoquinones was shown in the tests in which PQ was used as a neurotoxin (Figure 1D). This may indicate that the studied compounds are generally more capable of inhibiting the formation of paraquat cations and capturing the superoxide radicals generated by PQ than blocking the oxidation of 6-OHDA and the production of para-quinone and ROS.

However, despite the high antiradical activity of the studied basic 1,4-naphthoquinones, the mechanism of their protective action on cells can be complex. It is possible that the tested 1,4-NQs are additionally able to regulate the activity of enzymes participating in maintaining the redox balance in cells. On the one hand, they can activate a number of antioxidant enzymes, eliminating excess ROS in cells (superoxide dismutase, catalase, and peroxiredoxins). On the other hand, this is the inhibition of enzymes involved in the process of ROS generation, such as NADPH oxidase, xanthine oxidase, mitochondrial cytochrome c oxidase, and microsomal monooxygenases.

Moreover, it was already established that certain 1,4-NQs stimulate Nrf2-dependent activation of ARE-dependent gene expression followed by enhanced formation of cytoprotective proteins, such as antioxidant enzymes. Thus, 5-hydroxy-2-methyl-1,4-naphthoquinone (plumbagin) and 5,8-dihydroxy-1,4-naphthoquinone (naphthazarin) at subtoxic concentrations were found to stimulate Nrf2/ARE signalling and enhance the expression of Keap1/Nrf2/ARE-dependent target genes in HepG2 cells, human SH-SY5Y neuroblastoma cells, and primary rat cortical neurons. Exposure of neuronal cells to plumbagin provided protection against subsequent oxidative and metabolic insults, and administration of 1,4-NQ significantly reduced the amount of brain damage and ameliorated associated neurological deficits in a mouse model of focal ischaemic stroke [22,23]. Therefore, it cannot be ruled out that the tested synthetic 1,4-NQs at nanomolar subtoxic concentrations can not only act as free radical scavengers but also significantly activate the Nrf2/ARE signalling pathway, which is a key mediator of oxidative stress in cells. This requires further investigation. The proposed cytoprotective mechanism of 1,4-NQs against the neurotoxic action of PQ and 6-OHDA is displayed in Scheme 1.

For the compounds **U-572** and **U-623**, scavenging percentages were found to decrease with increase in their concentration. It is currently known that many quinones exhibit ambivalent effects on biological systems and have a U-shaped dose–response curve. Depending on the dose used, cellular targets and time, the action of quinones on cells results in toxicity or cytoprotection. At low doses, stable and selective quinones exhibiting cytoprotective properties come from electrophilic counterattacks, resulting in the induction of detoxification enzymes through the Keap1/Nrf2/ARE pathway and due to scavenging activity. At higher doses, less selective and more reactive quinones exhibit cytotoxic properties by inhibiting cell proliferation due to the formation of a semiquinone radical and superoxide, which can generate highly toxic hydroxyl radicals causing cell death [24]. Obviously, the more pronounced manifestation of scavenging activity at the lowest doses for a number of 1,4-naphthoquinones we found is explained by their U-shaped dose–response curve of action.

In our in silico investigation of 1,4-NQ derivatives, the SlogP_VSA (Log of the octanol/water partition coefficient) and SMR_VSA (Molecular refractivity) descriptors were the most important, describing the cytoprotective properties of naphthoquinones and correlated with their neuroprotective activity. These subdivided surface area descriptors (or VSA descriptors) are based on an approximate accessible van der Waals surface area (in Å2) calculation for each atom, vi, along with another atomic property, pi. SlogP_VSA is intended to capture hydrophobic and hydrophilic effects either in the receptor or on the way to the receptor, while SMR-VSA is intended to capture polarizability. Therefore, VSA descriptors represent the hydrophobicity, polarizability, and electrostatic profile of molecules, and these properties are indeed relevant to receptor affinity and ligand recognition, drug transport, and pharmacokinetics [25,26]. Another two important descriptors are TPSA and Vsurf descriptors. The topological molecular polar surface area (TPSA), that is, the surface belonging to polar atoms, is a descriptor that correlates well with the passive transport of molecules across membranes and, therefore, allows predicting the transport properties of drugs. This descriptor is widely used to rapidly screen for bioavailability of virtual libraries containing millions of molecules, absorption, cell membrane penetration, and crossing the blood–brain barrier. Vsurf descriptors depend on the structure connectivity and conformation. These descriptors have been shown to be useful in sorption and pharmacokinetic property prediction [27,28]. We applied an electrostatic map method to predict the electrostatically preferred locations of hydrophobic acceptor hydrogen bonds and hydrogen bond donors in the molecules of the two main 1,4-naphthoquinones. It is known that this nonlinear PBE methodology more accurately models interactions with hypothetical ligands and receptors compared to linear solvent screening techniques [29]. Using the PBE approach, it was demonstrated that the most effective molecule from the series of basic 1,4-naphthoquinones, **U-623**, has a large hydrophobic area and the presence of a significant number of acceptor sites in the molecule, which probably has a significant impact on the manifestation of neuroprotective action by this compound, especially in the case of the neurotoxin PQ. Thus, the obtained significant correlation between the neuroprotective properties of 1,4-NQs and the peculiarities describing the physicochemical properties of these compounds may indicate that the hydrophobicity, polarity, charge, and shape of the molecule can also be of decisive importance in the manifestation of the biological activity of the studied substances.

## 4. Material and Methods

### 4.1. Synthesis of 1,4-Naphtoquinones

All studied 1,4-naphtoquinones were synthesized according to the protocols described in [14]. The chemical structure and purity of the compounds were confirmed by HPLC, NMR spectroscopy, and mass-spectrometry.

### 4.2. Cell Line and Culture Condition

The murine neuroblastoma cell line Neuro-2a was purchased from ATCC (CCL-131™; American Type Culture Collection, Manassas, VA, USA). The cells were cultured in DMEM medium (Biolot, St. Petersburg, Russia) containing 10% fetal bovine serum (Biolot, St. Petersburg, Russia) and 1% penicillin/streptomycin (Biolot, St. Petersburg, Russia). Cells were incubated at 37 °C in a humidified atmosphere containing 5% (*v*/*v*) CO_2_.

### 4.3. Paraquat and 6-OHDA Induced In Vitro Model of Neurotoxicity

After 24 h of adhesion, Neuro-2a cells (1 × 10^4^ cells/well) were treated with compounds at concentrations of 0.01–1.0 μM for 1 h, after that 1.0 mM paraquat or 25.0–90.0 μM of 6-hydroxydopamine dissolved in H_2_O were added. Cells incubated without inductors or with inductors were used as corresponding positive and negative controls. Cell viability was measured after 24 h [30].

### 4.4. Cell Viability Assay

#### 4.4.1. MTT Test

Stock solutions of substances were prepared in DMSO at a concentration of 10.0 mM. All tested compounds were added to the wells of the plates in a volume of 20 μL diluted in PBS in final concentrations of 0.01, 0.1, or 1.0 μM.

Neuro-2a cells (1 × 10^4^ cells/well) were incubated in a CO_2_ incubator for 24 h at 37 °C for adhesion. After that, 20 μL of test solution were loaded to the cells and incubated for 24 h. After incubation, the medium with tested substances was replaced by 100 μL of fresh medium. Then, 10 μL of MTT (3-(4,5-dimethylthiazol-2-yl)-2,5-diphenyltetrazolium bromide) (Sigma-Aldrich, St. Louis, MO, USA) stock solution (5 mg/mL) was added to each well and the microplate was incubated for 4 h. After that, 100 μL of SDS-HCl solution (1 g SDS/10 mL dH_2_O/17 μL 6 N HCl) was added to each well followed by incubation for 18 h. The absorbance of the converted dye formazan was measured using a Multiskan FC microplate photometer (Thermo Scientific, Waltham, MA, USA) at a wavelength of 570 nm [31]. All experiments were repeated in triplicate. Cytotoxic activity was expressed as the percent of cell viability.

#### 4.4.2. FDA Staining

A stock solution of the fluorescein diacetate (FDA) (Sigma-Aldrich, St. Louis, MO, USA) in DMSO (1 mg/mL) was prepared. The cells (1 × 10^4^ cells/well) were seeded in 96-well plates before testing. After incubation of the cells with compounds and PQ or 6-OHDA during 24 h, FDA solution (50 μg/mL) was added to each well and the plate was incubated in the dark at 37 °C for 15 min. Cells were washed with PBS, and fluorescence was measured with a PHERAstar FS plate reader (BMG Labtech, Ortenberg, Germany) at λ_ex_ = 485 nm and λ_em_ = 518 nm. The data were processed by MARS Data Analysis v. 3.01R2 (BMG Labtech, Ortenberg, Germany). Cell viability was expressed as the percent of control [32].

#### 4.4.3. PI Staining

After 24 h of adhesion, Neuro-2a cells (5 × 10^4^ cells/mL) in 24-well plate were treated with compounds at concentrations of 0.01–1.0 μM for 1 h, after that 1.0 mM paraquat or 25.0 μM of 6-hydroxydopamine were added and incubate with cell for 24 h. After incubation, cells were trypsinized, harvested, and washed twice with PBS. Propidium iodide (Sigma-Aldrich, St. Louis, MO, USA) was added to the cell suspension at 10 μg/mL in H_2_O (final concentration), incubated for 3 min in the dark, and the fluorescence was measured by the Muse™ Cell Analyzer (EMD Milipore Corp., Temecula, CA, USA). All experiments were repeated in triplicate. Cytotoxic activity was expressed as the percent of cell viability [33].

### 4.5. Cell Cycle Analysis

The cell cycle analysis was performed as recently described [34]. Briefly, Neuro-2a cells (5 × 10^4^ cells/mL) exponentially growing in 12-well plates were treated with PQ (1.0 mM) or 6-OHDA (25.0 μM) and different concentrations of 1,4-NQs (0.01–1.0 μM) for 24 h. After incubation, cells were trypsinized, harvested, washed with PBS, and fixed with ice-cold 70% ethanol in a dropwise manner prior to storage at −20 °C overnight. The cells were then washed with PBS, incubated with 200 μg/mL RNAse and 20 μg/mL of PI for 30 min at 37 °C and the DNA content was analyzed by the Muse™ Cell Analyzer (EMD Milipore Corp., Temecula, CA, USA). The proportion of cells in each phase of the cell cycle was expressed as a percentage.

### 4.6. Mitochondrial Membrane Potential (MMP) Detection

After 24 h of adhesion, the cells (1 × 10^4^ cells/well of a 96-well plate) were incubated with different concentrations of 1,4-NQs (0.01–1.0 μM) for 1 h. Then, PQs or 6-OHDAs were added to cell to for incubation during 1 h or 5 h, respectively. The cells incubated without PQ or 6-OHDA and compounds and with PQ or 6-OHDA alone were used as positive and negative controls, respectively. The tetramethylrhodamine methyl (TMRM) (Sigma-Aldrich, St. Louis, MO, USA) solution at 500 nM was added in each well, and the cells were incubated for 30 min at 37 °C. The intensity of fluorescence was measured with a PHERAstar FS plate reader (BMG Labtech, Ortenberg, Germany) at λ_ex_ = 540 nm and λ_em_ = 590 nm. The data were processed by MARS Data Analysis v. 3.01R2 (BMG Labtech, Ortenberg, Germany). The results were presented as a percentage of positive control data [32].

### 4.7. ROS and NO Level Analysis

After 24 h of adhesion, Neuro-2a cells (1 × 10^4^ cells/well) were incubated with compounds at concentrations of 0.01–1.0 µM for 1 h. Then, PQ at a concentration of 1.0 mM or 6-OHDA at concentration 25.0 μM were added in each well, and cells were incubated for 1 h or 3 h, respectively. In order to study ROS formation, 20 µL of 2,7-dichlorodihydrofluorescein diacetate solution (10.0 µM H2DCF-DA, Molecular Probes, Eugene, OR, USA) was added to each well such that the final concentration was 10.0 µM, and the microplate was incubated for an additional 10 min at 37 °C in the dark.

In the case of determining the NO production, a DAF-FM fluorescent probe solution (5.0 μM, Molecular Probes, Eugene, OR, USA) was added to each well, the microplate was further incubated for 40 min at 37 °C in the dark. In both cases, the fluorescence intensity was measured using a high-speed plate reader PHERAstar FS (BMG Labtech, Ortenberg, Germany) at λ_ex_ = 485 nm and λ_em_ = 518 nm. The data were processed by MARS Data Analysis v. 3.01R2 (BMG Labtech, Ortenberg, Germany). The results were presented as a percentage of positive control data [35].

### 4.8. Radical Scavenging Assay

DPPH radical scavenging activity of compounds was tested as described [36] with minor modifications. The compounds were dissolved in MeOH, and the solutions 1,4-NQs or ascorbic acid (Vekton, St. Petersburg, Russia) as a positive control (120 μL) were dispensed into wells of a 96-well microplate. In all of them, 30 μL of the DPPH (Sigma-Aldrich, Steinheim, Germany) solution in MeOH (0.75 mM) was added to each well. The concentrations of compounds and ascorbic acid in mixture were 0.01–1.0 μM. The plates were incubated in the dark at room temperature for 30 min, and then the absorbance was measured at 517 nm with a Multiskan FC microplate photometer (Thermo Scientific, Waltham, MA, USA). The negative control contained no test compound. The final results were reported as ED_50_, which is the concentration of compound that scavenged 50% of DPPH radicals in the reaction solution.

### 4.9. Computer Modeling and Quantitative Structure-Activity Relationship (QSAR) Analysis

The dataset of the most active basic 1,4-NQs (Table 1 and Figure 1) was used for 3D-structure modeling and optimization with Amber10:EHT force field using the Build module of the MOE 2020.09 program (Montreal, QC, Canada) [29]. The MOE database of energy-minimized 3D-structures of 1,4-NQs derivatives was used to calculate descriptors using the QuaSAR MOE 2020.09 module. The values of the neuroprotection value of 1,4-NQs in PD models Neuro-2a cell induced by PQ and 6-OHDA were expressed in % relative to the effect of the inducer alone.

## 5. Conclusions

Our results indicate that among the 1,4-naphthoquinones and their derivatives synthesized by us and composing a combinatorial mini-library, compounds with pronounced neuroprotective properties were found. A number of selected compounds were capable of protecting neuronal cells both when they were incubated with paraquat and with 6-OHDA. This is reflected in the ability of these compounds to protect Neuro-2a cells from death, to normalize the cell cycle, to restore mitochondrial function and mitochondrial membrane potential, and to reduce ROS levels. Since the molecular mechanism damaging cells is practically the same for both neurotoxins used, it can be assumed that the mechanism of the protective action of the selected 1,4-NQs also has similar features. The pronounced ability of the most active compounds to capture free radicals, which we discovered, is obviously a key link in their neuroprotective mechanism. It consists of protecting cells from oxidative stress generated by both neurotoxins. The most active neuroprotectors were found among the basic 1,4-NQs. The QSAR study clearly showed that the physicochemical properties of basic 1,4-NQs, such as hydrophobicity, polarizability, and electrostatic profile of molecules, can play a key role in exerting biological effects and provide better and faster sorption and penetration of these compounds into cells through biomembranes. Further studies of the ability of these 1,4-naphthoquinones to maintain the redox potential of neuronal cells by regulating the activity of the enzymatic orchestra responsible for the balance of reactive oxygen species together with the investigation of their ability to penetrate biological barriers will help to establish a more detailed molecular mechanism of the neuroprotective action of these 1,4-naphthoquinones.

## Figures and Tables

**Table 1:** Chemical structures of studied 5,8-dihydroxy-1,4-naphthoquinone derivatives.

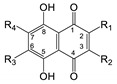						
#	**Code**	**R_1_**	**R_2_**	**R_3_**	**R_4_**	EC_50_, μM *
	**1. Basic naphthoquinones**					
1	U-193	H	H	H	H	>100
2	U-139	HO	H	H	H	>100
3	U-574	HO	Me	H	H	>100
4	U-575	HO	Et	H	H	>100
5	U-134	HO	HO	H	H	>100
6	U-572	HO	HO	Me	H	>100
7	U-573	HO	HO	Et	H	>100
8	U-138	HO	HO	Et	HO	>100
9	U-195	HO	H	Cl	Cl	23.10
10	U-434	HO	H	Me	Me	82.75
11	U-622	HO	H	H	MeO	>100
12	U-623	HO	H	MeO	MeO	>100
13	U-504	HO	H	HO	HO	>100
14	U-642	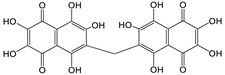				>100
15	U-643	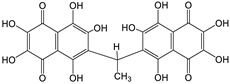				>100
	**2. Acetylated naphthoquinone O-glucosides**					
16	U-127	AGO	H	H	H	16.43
17	U-132	GO	H	H	H	87.40
18	U-444	AGO	Me	H	H	5.33
19	U-443	AGO	Et	H	H	4.46
20	U-330	AGO	H	Me	Me	9.43
21	U-136	AGO	AGO	H	H	10.60
22	U-420	AGO	AGO	Me	H	4.46
23	U-421	AGO	AGO	Et	H	8.84
24	U-133	AGO	AGO	Et	AGO	8.45
	**3. Acetylated naphthoquinone S-glucosides**					
25	U-633	HO	AGSCH_2_	H	H	>100
26	U-519	HO	AGSCH_2_	Me	Me	84.00
27	U-518	HO	AGSCH_2_	Cl	Cl	32.20
28	U-639	HO	AGSCH_2_	H	MeO	>100
29	U-637	HO	AGSCH_2_	MeO	MeO	>100
30	U-634	MeO	AGSCH_2_	H	H	2.72
31	U-521	MeO	AGSCH_2_	Me	Me	>100
32	U-523	MeO	AGSCH_2_	Cl	Cl	3.14
33	U-645	MeO	AGSCH_2_	H	MeO	11.61
34	U-638	MeO	AGSCH_2_	MeO	MeO	11.05
	**4. Deacetylated naphthoquinone S-glucosides**					
35	U-635	HO	GSCH_2_	H	H	>100
36	U-520	HO	GSCH_2_	Me	Me	>100
37	U-624	HO	GSCH_2_	Cl	Cl	>100
38	U-644	HO	GSCH_2_	H	MeO	>100
39	U-640	HO	GSCH_2_	MeO	MeO	>100
40	U-636	MeO	GSCH_2_	H	H	19.02
41	U-522	MeO	GSCH_2_	Me	Me	11.47
42	U-625	MeO	GSCH_2_	Cl	Cl	38.02
43	U-646	MeO	GSCH_2_	H	MeO	80.76
44	U-641	MeO	GSCH_2_	MeO	MeO	61.60
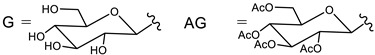						

* Polonik et al. *Mar. Drugs* **2020**, *18*, 602; doi:10.3390/md18120602 [14].

**Table 2:** Free radical scavenger capacity of selected 5,8-dihydroxy-1,4-naphthoquinone derivatives.

Code	Scavenging of DPPH, %		
	0.01 µM	0.1 µM	1.0 µM
U-134	14.55 ± 1.30 *	16.87 ± 3.57 *	30.85 ± 3.57 *
U-572	18.95 ± 0.52 *	16.13 ± 2.03 *	28.22 ± 1.16 *
U-623	16.08 ± 2.79 *	15.46 ± 1.09 *	30.34 ± 4.11 *
U-643	6.52 ± 0.02 *	14.04 ± 2.73 *	21.87 ± 1.01 *
U-625	9.57 ± 0.94 *	14.29 ± 0.45 *	30.25 ± 0.55 *
Ascorbic acid	4.23 ± 0.16	6.59 ± 0.27	47.98 ± 3.48

* *p* < 0.05 compared to ascorbic acid.

**Table 3:** Description of the molecular descriptors used for QSAR model generation for the neuroprotective activity of 1,4-NQs.

Class	Code	Description
2D	SMR_VSA	Molecular refractivity (including implicit hydrogens). This property is an atomic contribution that assumes the correct protonation state (washed structures). Sum of vi such that Ri is in [0, 0.11].
2D	SlogP_VSA	Log of the octanol/water partition coefficient (including implicit hydrogens). This property is an atomic contribution model that calculates logP from the given structure; i.e., the correct protonation state). Sum of vi such that Li is in [−0.2, 0].
2D	TPSA	Topological polar surface area (Å^2^) calculated using group contributions to approximate the polar surface area from connection table information only.
3D	vsurf_D	Hydrophobic volume.

## Data Availability

The data presented in this study are available upon request from the corresponding author.

## Abbreviations

1:4-NQs	1,4-Naphthoquinones
6-OHDA	6-Hydroxydopamine
ARE	Antioxidant responsive element
DAF-FM	4-Amino-5-Methylamino-2′,7′-Difluorofluorescein Diacetate
DMEM	Dulbecco’s Modified Eagle Medium
DMSO	Dimethylsulfoxide
DPPH	2,2-Diphenyl-1-picrylhydrazyl
FDA	Fluorescein diacetate
H2DCF-DA	2,7-Dichlorodihydrofluorescein diacetate
Keap1	Kelch-like ECH-associated protein 1
MAO	Monoamine oxidase
MMP	Mitochondrial membrane potential
MPTP	1-Methyl-4-phenyl-1,2,3,6-tetrahydropyridine
MTT	3-(4,5-Dimethylthiazol-2-yl)-2,5-diphenyltetrazolium bromide
NADPH	Nicotinamide adenine dinucleotide phosphate
NO	Nitric oxide
Nrf2	Nuclear factor E2-related factor 2
PBS	Phosphate-buffered saline
PD	Parkinson’s disease
PI	Propidium iodide
PQ	Paraquat or *N*,*N*′-dimethyl-4,4′-dipyridylium dichloride
QSAR	Quantitative structure-activity relationship
ROS	Reactive oxygen species
SDS	Sodium dodecyl sulfate
TMRM	Tetramethylrhodamine methyl ester perchlorate
